# Ex vivo inhibition of enamel phosphate uptake in Rothia dentocariosa

**DOI:** 10.1099/jmm.0.002192

**Published:** 2026-08-07

**Authors:** Dhiraj Kumar, Robert S. Jones, Beverly E. Flood, Jake V. Bailey

**Affiliations:** 1 Department of Diagnostic & Biological Sciences, University of Minnesota, Minneapolis, MN 55455, USA; 2 Department of Developmental & Surgical Sciences, University of Minnesota, Minneapolis, MN 55455, USA; 3 Department of Earth and Environmental Sciences, University of Minnesota, Minneapolis, MN 55455, USA

**Keywords:** bacteria, dental caries, phosphorus radioisotopes, polyphosphate, tooth remineralization

## Abstract

**Introduction.** Oral bacteria, such as *Rothia dentocariosa* (*Rd*), can modulate external aqueous phase orthophosphate (P_i_) concentrations via P_i_ internalization and accumulate high amounts of P_i_ in the form of polyphosphate (polyP).

**Hypothesis/Gap Statement.** This study investigates whether the carbohydrate metabolism of *Rd* that generates acid and solubilizes P_i_, derived from phosphorus-32 (^32^P)-labelled tooth enamel, can be internalized and incorporated into intracellular polyP. The study investigated the hypothesis that the internalization of tooth-derived P_i_ and polyP synthesis could be attenuated.

**Aim.** A radionuclide ^32^P-labelled remineralized model examined the influence of gallein, an inhibitor of polyP kinases, on the ingestion of tooth-derived P_i_ and on the bacterium’s cultivability.

**Methodology.** Radionuclide ^32^P-labelled remineralized bovine enamel (*n*=6) was exposed to *Rd* (ATCC 17931) incubated in brain heart infusion (BHI) broth containing glucose. Attenuated total reflectance Fourier transform infrared (FTIR) spectroscopy and cross-polarization optical coherence tomography assessed the surface and subsurface characteristics of the remineralized and demineralized enamel surface. Liquid scintillation traced the ^32^P release from the tooth to the intracellular polyP in gallein^pos/neg^ samples. Cell-media, supernatant, cell resuspension, and extracted polyP, from *Rd* exposed to the radioisotope-labelled remineralized enamel, were analysed for ^32^P activity at 10 and 22 h time points.

**Results.** FTIR determined that ^32^P was incorporated in dental enamel lesions and was released after exposure to an acidic buffer (pH=4.5) or *Rd*. After 24 h of exposure, gallein^pos^ reduced *Rd*’s planktonic growth and increased pH by 7.45% and 7.19%, respectively, compared to gallein^neg^. Gallein inhibited the cultivability of planktonic *Rd* on BHI agar plates with a 13- to 60-fold reduction of c.f.u. ml^−1^ (25 µM–13.03×, 50 µM–19.58× and 100 µM–60.57×) compared to gallein^neg^ samples. Isotope labelling demonstrated that *Rd* used P_i_ derived directly from the tooth for the benefit of the bacterium’s fitness through the formation of polyP.

**Conclusion.** *Rd* used the P_i_ derived directly from the tooth for the benefit of the bacterium’s fitness. Gallein attenuated polyP accumulation, reduced bacterial cultivability and may be a potential therapy option in the prevention and treatment of primary and secondary caries.

## Data Summary

The data that support the findings are available in the searchable Data Repository for the University of Minnesota (https://hdl.handle.net/11299/276448). The reference sequence for *Rothia dentocariosa* (ATCC 17931) can be found in the National Center for Biotechnology Information (NC_014643.1).

## Introduction

Oral bacteria such as *Rothia dentocariosa* (*Rd*) can modulate external aqueous phase orthophosphate (P_i_) concentrations via P_i_ internalization and accumulate high amounts of P_i_ in the form of a long-chain polyanion, polyphosphate (polyP) [1]. The metabolic utility of polyP is related to the modulation of stress response [2]. PolyP can serve as an energy reserve and stress response regulator during nutrient limitations and surface colonization [3]. In assessing annotated genomes of the oral microbiome [4, 5], numerous oral bacteria possess the genes known to encode enzymes capable of polyP synthesis (polyP kinases ppk1 and ppk2) and polyP hydrolysis (exopolyphosphatase ppx) [6, 7]. Our previous work demonstrated that *Rd* is a useful model organism when considering the potential effects of P_i_ modulation and polyP accumulation in oral environments [1]. From the results of our previous work, it was postulated that the ‘luxury uptake’ of P_i_ might be an added factor of cariogenicity in *Rd* since it may affect bacterial survival and viability. As inferred from its taxonomic nomenclature, *Rd* was first isolated and is often associated with caries [8]. As is the case for most phylotypes in the oral microbiome, environmental factors drive phenotype expression. *Rothia* spp. can be associated with both disease and health outcomes [9–11]. Importantly, *Rothia* spp. are also frequently found in relatively high abundance in dental plaque with *Rd* as a prominent initial colonizing phylotype on dental hard tissue [12, 13].

In the present study, a novel enamel demineralization model was developed to test whether a bacterium with polyP metabolism uptakes P_i_ derived not only from surrounding media but from phosphate molecules that were previously incorporated into the enamel hydroxyapatite (HA) crystal. In the 1960s and 1970s, Heikki Luoma established the experimental evidence that tooth-derived P_i_, measured with the aid of a phosphorus (P) radioisotope [phosphorus-32 (^32^P)], could be internalized into *Streptococcus mutans* (strain FA-1) and the magnitude of absorbed ^32^P was associated with a substantial pH decrease [14–16]. This groundbreaking work did not investigate the molecular fate of internalized ^32^P. Nor did Luoma’s work lead to future investigations in other bacterial species. This may be partly due to the narrow focus of studying *S. mutans* in caries research for several decades based on its acidogenic, aciduric and adhesion properties [17, 18]. While *S. mutans* may take up some P_i_, it has been demonstrated recently that the level of P_i_ uptake is substantially lower than measured in an oral bacterium, *Corynebacterium matruchotii*, that accumulates P_i_ and can synthesize P_i_ into the storage molecule polyP [7]. With the near-widespread acceptance of the plaque ecology hypothesis and an appreciation for the complex metabolism of the full oral microbiome, there is a new opportunity to revisit the research question on the effects of P_i_ uptake, especially those within dental plaque that possess the canonical genes for polyP metabolism.

This study investigates whether the carbohydrate metabolism of *Rd* that generates acid and solubilizes P_i_, derived from ^32^P-labelled tooth enamel, can be internalized and incorporated into intracellular polyP. The study also examined if this internalization of tooth-derived P (inorganic phosphate) could be attenuated with gallein, an inhibitor of polyP kinases. Through a reduction of polyP, gallein has the potential to limit polyP accumulation of tooth-derived P and reduce the viability of *Rd*. The present study is relevant to understanding how P derived directly from the tooth enamel may contribute to the viability of *Rd*. Since the human genome does not contain polyP kinases, the investigation of the effects of gallein on the viability of planktonic *Rd* to form direct surface colonies has clinical relevance. Gallein treatment may be a potential therapy option in the prevention and treatment of primary and secondary caries.

## Methods

### *In vitro* enamel demineralization

Anterior bovine teeth (*n*=6) were collected post-mortem from a local meat-packing company, cleaned, sectioned into enamel crown fragments (10 mm × 10 mm) and partially embedded in a polymer resin. Baseline optical reflectivity images were obtained by scanning the enamel samples with the shortwave infrared (SWIR) cross-polarization optical coherence tomography (CP-OCT) as described in Material S1 (available in the online Supplementary Material) (supplemental methods, reflectivity and optical coherence tomography). Artificial enamel subsurface demineralization was achieved with the use of an acidic buffer solution saturated with calcium and phosphate with respect to HA. Bovine enamel was exposed for 5 days at 37 °C to a demineralizing buffer solution (pH=4.5) with 0.075 mol l^−1^ acetate, 2.0 mmol l^−1^ calcium and 2.0 mmol l^−1^ phosphate. All glassware was cleaned and washed using a near-neutral pH and phosphate-free detergent (Neutrad, Decon Labs Inc., PA) and rinsed with commercially available double distilled water.

### P isotope and *in vitro* enamel remineralization

**Fig. 1.**
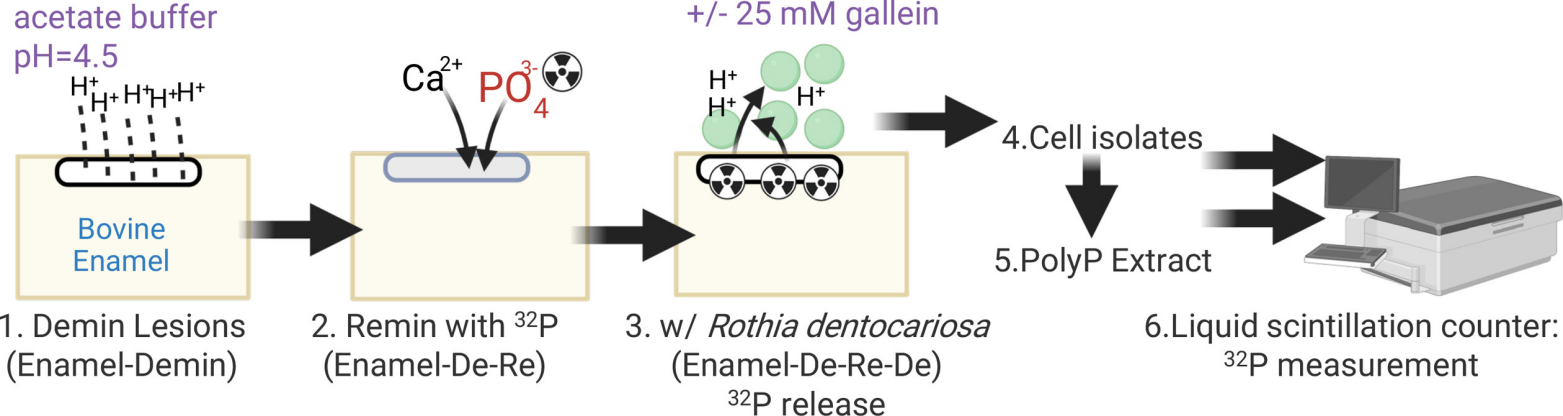
Enamel caries model with ^32^P radioisotope where (1) artificial lesions are created by an acetate buffer on enamel sections embedded in polymer blocks; (2) artificial lesions are exposed to a remineralization solution that contains ^32^P that gets incorporated into the dental enamel; (3) the ^32^P-labelled enamel is exposed to *Rothia dentocariosa* with and without 25 mM of gallein; (4) at 10 and 22 h, bacteria are isolated; (5) polyP inclusions were extracted from the bacterial cells; and (6) beta-emission from ^32^P was measured.

After artificial lesions were created and scanned with CP-OCT, these enamel samples were remineralized (remineralizing solution recipe in SI) in the presence of monopotassium phosphate (KH_2_PO_4_) with and without a ^32^P radionuclide. Fig. 1 shows the schematics for the steps leading to demineralization, remineralization (w/wo ^32^P) and demineralization (w/wo bacteria) and data collection using scintillation counts. Initially, monopotassium phosphate, with ^32^P radionuclide (1 mCi), in 1 ml was ordered and received (day 0) through the department of radiation safety. ^32^P radionuclide experiments occurred in an enclosed room with proper plastic shielding for safety and prevention of cross-contamination. A known volume of ^32^P solution was mixed with deionized water, and serial dilution was used to prepare different concentrations of ^32^P; from each dilution, a known volume was mixed with 10.0 ml liquid scintillation cocktail solution (Revvity Health Sciences Inc Ultima Gold cocktail, NC0169557, Fisher Scientific) in a 1 : 10 ratio. Beta-emission was measured by a liquid scintillation counter (LS6500, Beckman Coulter, Brea, CA, USA). To calibrate the ^32^P beta-emission decay per time, standardized calibration curves were created by measuring KH_2_PO_4_ (ng ml^−1^) against scintillation counts over the course of 6 days (Fig. S1).

Following the creation of artificial lesions, demineralized samples were exposed from day 0 to day 3 to a 10-ml remineralizing solution [without or with ^32^P, 30 µCi (30 µl in 10 ml)] with a 20 mmol l^−1^ cacodylate buffer (pH=7) at 37 °C with 1.5 mmol l^−1^ calcium, 0.9 mmol l^−1^ phosphate and 150 mmol l^−1^ KCl. This remineralization protocol was initially analysed (*n*=6) with CP-OCT to assess for non-HA calcium phosphate surface and subsurface precipitation. Attenuated total reflectance Fourier transform infrared (ATR-FTIR) spectroscopy assessed the surface characteristics of the remineralization enamel surface. The FTIR spectrum was collected from 400 to 4,000 cm^−1^. This was performed in reflectance mode, 30 scans per sample, 2 cm^−1^ resolution. The collected IR spectrum was analysed using the OMNIC software (Thermo Fisher, Waltham, MA) on a Nicolet iS50 FTIR system. The remineralization protocol was performed for the remainder of the samples with a trace amount of ^32^P monopotassium phosphate diluted to 30 µCi activity per sample solution. An initial assessment of the remineralization potential of ^32^P derived from monopotassium phosphate on select samples (*n*=6) of the radionuclide ^32^P bovine enamel was exposed to neutral water and the original demineralization (pH=4.5) solution. This approach assessed the amount of ^32^P derived from monopotassium phosphate that was surface absorbed vs. incorporated into remineralized HA after 3 days.

### Bacterial exposure to ^32^P-labelled remineralized enamel

On day 3 of the experiment, the new radionuclide ^32^P-labelled remineralized bovine enamel (*n*=6) was exposed to *Rd* (ATCC 17931) incubated in brain heart infusion (BHI) broth containing glucose, 2 g l^−1^ (#53286, Millipore Sigma, Burlington, MA, USA). Prior to incubation, *Rd* cells were grown on BHI medium-agar plates followed by transfer of 1–2 colonies to 5.0 ml BHI broth and kept under aerobic conditions overnight at 37 ℃ with continuous shaking (180 r.p.m.). The bacterial cells were transferred into new BHI broth with an initial optical density (OD) of ~0.15 (2.3×10^8^ c.f.u. ml^−1^). The ^32^P-labelled remineralized enamel samples were added to the bacteria/BHI broth and kept under aerobic conditions for 22 h with continuous shaking (180 r.p.m.). Gallein (C_20_H_12_O_7_.2 (1/2)H_2_O; Molecular Weight (MW), 409.35 g mol^−1^; Chemical Abstracts Service (CAS), 2103-64-2, International Union of Pure and Applied Chemistry (IUPAC) name, 3′,4′,5′,6′-tetrahydroxyspiro[isobenzofuran-1(3 h),9′-(9 h)xanthen]−3-one) was purchased from Tocris Bioscience (Bristol, UK). At the start of incubation, samples were grown in the presence and absence of 25 mM gallein (gallein^pos/neg^). Bacterial growth and acidity were monitored by OD and pH. On day 4, the cell-media, supernatant, cell resuspension and extracted polyP from *Rd* exposed to the radioisotope-labelled remineralized enamel were analysed for ^32^P activity at 10 and 22 h time points as described in Material S1.

### Statistical analysis

Pairwise comparison of enamel reflectivity samples during demineralization and remineralization was analysed with a Tukey’s multiple comparison test after a one-way ANOVA (GraphPad Prism, Dotmatics, Boston, CA). Data sets comparing the effects of gallein versus controls were analysed with a one-way ANOVA with a Šídák’s multiple comparisons test (GraphPad Prism, Dotmatics, Boston, CA). Longitudinal growth and pH data were analysed with a repeated measures ANOVA. Data graphs were presented with means and 95% confidence intervals as error bars.

## Results

### Validation experiment

**Fig. 2.**
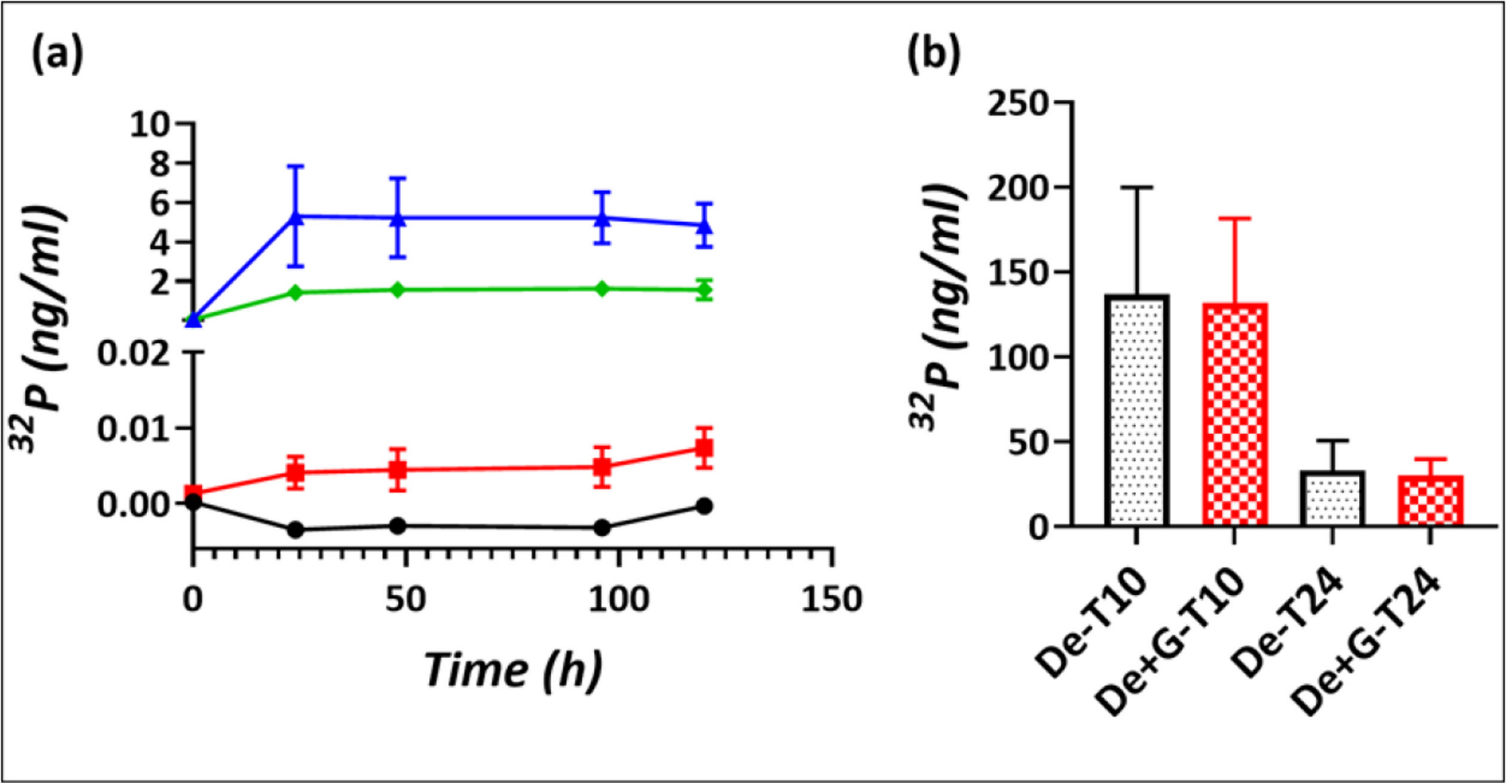
Testing the model (a) Enamel-De-Re with ^32^P was embedded in polymer blocks and exposed for 120 min with water (control: green line) or an acetate buffer solution (demin: blue line). The demin solution released ^32^P from the enamel substantially more than water. Almost no ^32^P was released from samples containing only polymer blocks (red line) and a sample holder with no sample (blank: black line). (b) De-T10 represents the measured ^32^P release after 10 h. De-T24 represents the release that occurs 14 h after the first 10 h. The presence of gallein (G) in the solution (De+G-T10; De+G-T24) did not change the release profile.

The validation experiment examined ^32^P absorbance into polymer blocks with and without demineralized enamel samples. Demineralized enamel sections, which were embedded in polymer blocks, were remineralized for 3 days in the presence of ^32^P radionuclide, monopotassium phosphate (De-Re^32^P). De-Re^32^P samples had substantially higher (*P*<0.05) release of ^32^P when exposed for 120 h to the acetate buffer solution (pH=4.5) than water (pH=7) (Fig. 2a). Uniform methacrylate polymer blocks (without embedded enamel sections) that were incubated in the presence of ^32^P radionuclide, monopotassium phosphate, for 3 days and transferred to a water solution had markedly less ^32^P release than the De-Re^32^P samples. In another experiment, De-Re^32^P samples were subjected to additional demineralization (De-Re^32^P-De) challenge by the demineralizing buffer with (gallein^pos^) and without (gallein^neg^) the presence of 25 µM gallein (Fig. 2b). Both De-Re^32^P-De gallein^pos/neg^ samples had similar levels of ^32^P release after 10 h (T10) and the subsequent 14 h (T24) exposure to the demineralization solution. There was higher release of ^32^P in the first 10 h (T10) than the subsequent 14 h (T24) for both gallein^pos^ and gallein^neg^. In the absence of bacteria, gallein did not have a direct effect on demineralization characteristics.

### Influence on *Rd* and mineralization

**Fig. 3.**
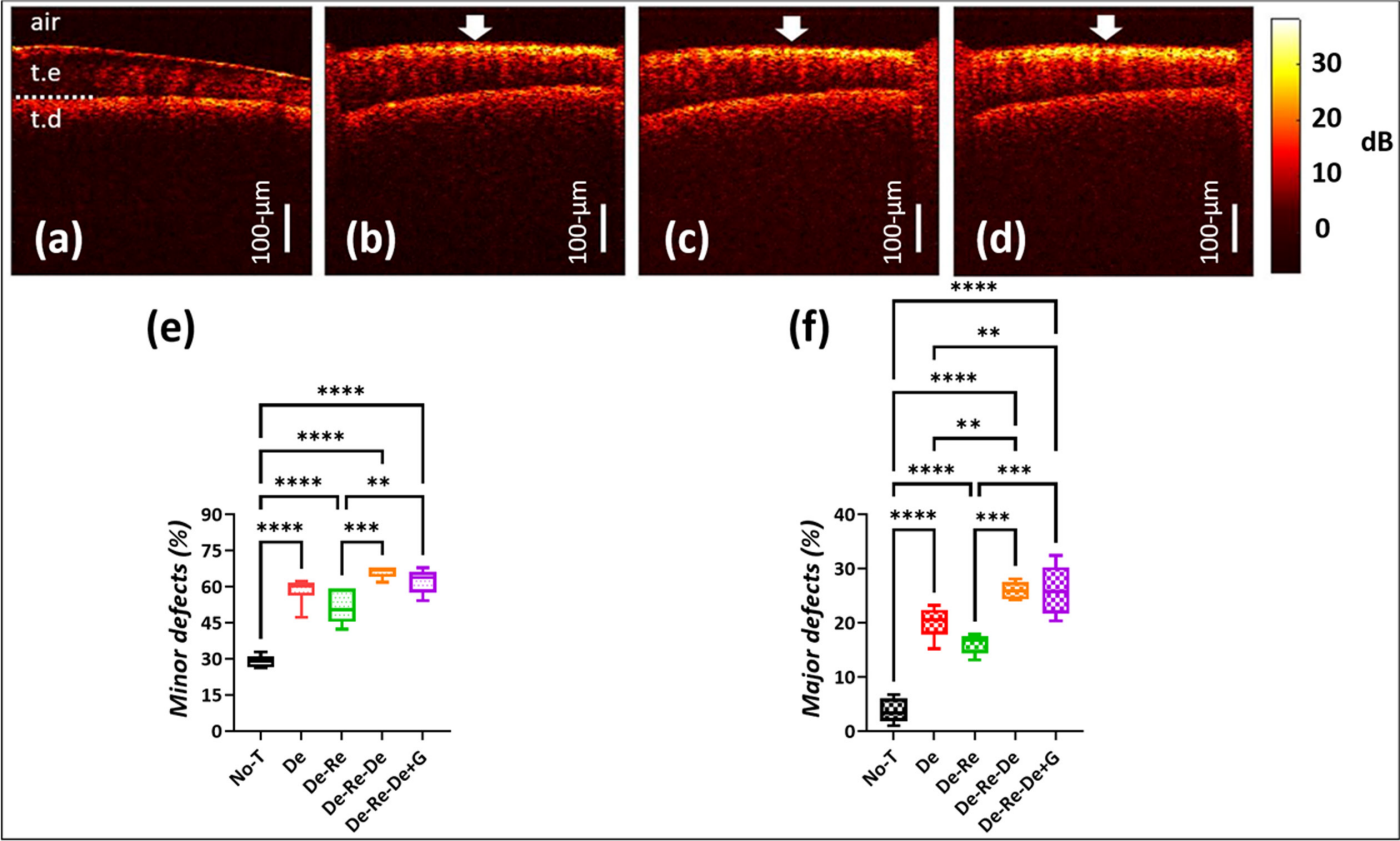
SWIR CP-OCT cross-sectional images representing backscattered reflectivity in a logarithmic [decibel (dB)] false color scale with white scale bars representing 100 µm of optical depth: (a) Tooth section where sound enamel (t.e) has minor backscattering, dentin–enamel junction is visualized (dotted line) and dentin (t.d) has higher scattering (No-T-control). (b) Enamel demineralization (Enamel-De) shows a subsurface lesion (arrow) with higher scattering. (c) After remineralization (Enamel-De-Re), the enamel has equivalent scattering. (d) Demineralization of the remineralized lesion (De-Re-De) shows higher scattering including with gallein (+G). Quantification of (e) minor-moderate and (f) major increases in scattering in demineralization defects. Moderate reflectivity changes at 2 dB above baseline are nearly a 50% increase in scattering. Major changes in reflectivity (≥20 dB above baseline) are close to a 100-fold increase and visualized as an orange/yellow signal with the false color scale in the CP-OCT images. ** for *P*≤0.01, ** for *P*≤0.001, *** for *P*≤0.0001.

SWIR reflectivity CP-OCT images measured and visualized the baseline reflectivity of sound enamel (Fig. 3a) and the increase of reflectivity after samples were exposed for 5 days to the demineralizing acetate buffer solutions (enamel–demin, Fig. 3b). After 3 days of remineralization (Enamel-De-Remin), CP-OCT images showed a consistent but non-significant trend of decreased reflectivity in moderate and major intensity versus initial abiotic artificial demineralization (Fig. 3c). The second phase of demineralization demonstrated that reflectivity did substantially increase, both in the presence and absence of gallein (De-Re-De samples (gallein^pos/neg^), when the enamel lesions were subjected to the additional *Rd* bacterial acidic challenge (Enamel-De-Re-De, Fig. 3d). Mineral dissolution was quantified with CP-OCT to assess the level of moderate (Fig. 3e) and high scattering defects (Fig. 3f).

**Fig. 4.**
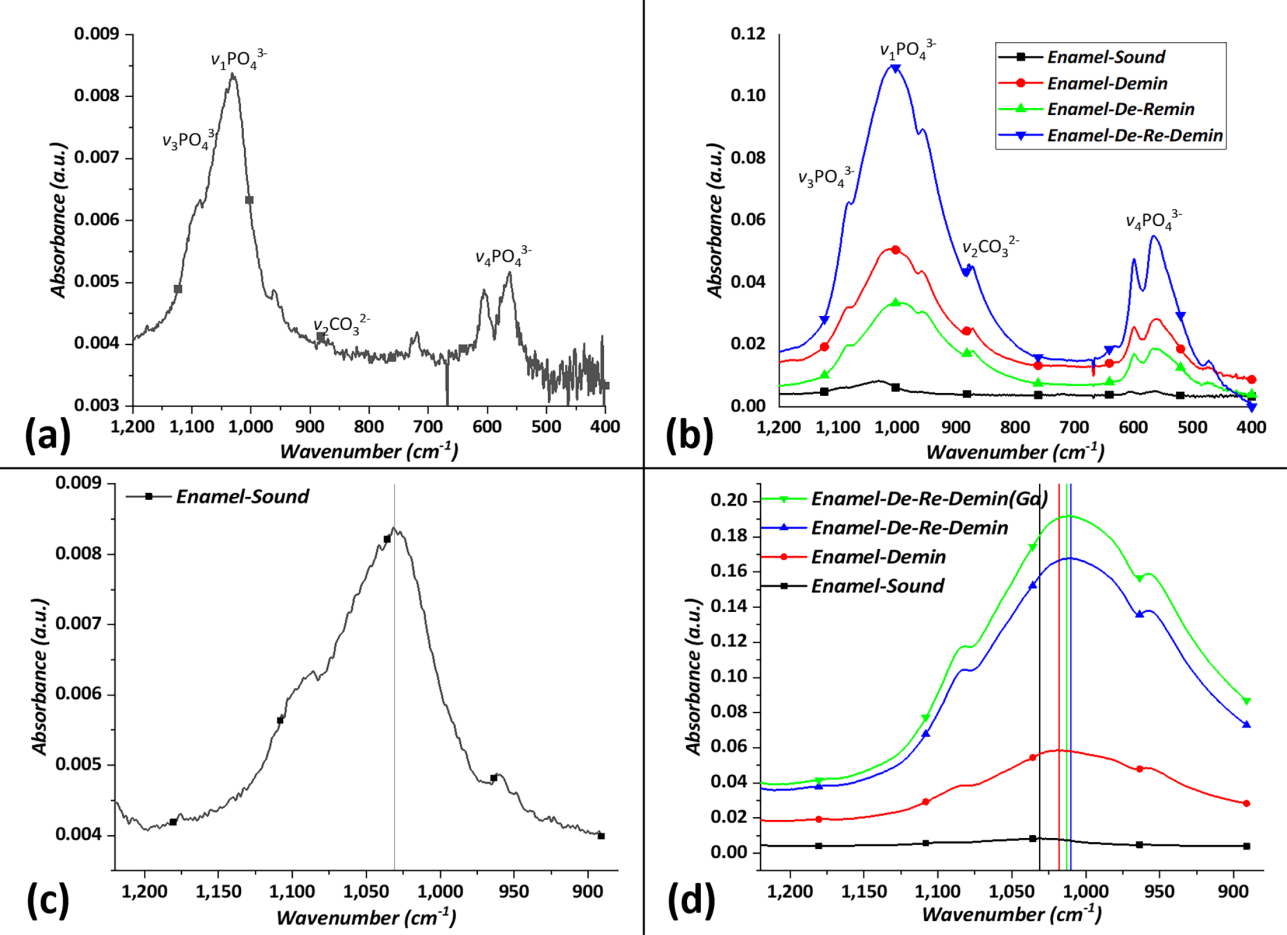
ATR-FTIR spectrum: (a) enamel NT; (b) enamel treatment samples; (c) enamel NT of the main stretching vibration of v1 PO43-, v4 PO43- and v2 C032-; and (d) center peak of v1 PO43- in treatment samples including *R. dentocariosa* samples with gallein (Ga).

The FTIR spectra of the no-treatment (NT) control enamel were similar to those measured in subsequent demineralization and remineralization challenges, with the exception that the signal intensity increased after further demineralization (Fig. S2). In the 400–1,200 cm^−1^ spectra of the dental enamel surface, the large band at 1,030 cm^−1^ corresponded to the stretching vibration of v1 PO with scissor vibrations of v4 PO_4_^3-^ at 525–610 cm^−1^ (Fig. 4a). The low shoulder band within the v1 PO peak near 870 cm^−1^ was caused by the v2 CO_3_^2-^ vibration. The spectral characteristics of the phosphate vibrations (v1 and v4) were nearly identical during the demineralization–remineralization–demineralization process (Fig. 4b). Throughout the demineralization and remineralization process, there was a minor shift of the asymmetrical stretch vibration (v3) of PO_4_, from 1,031 to 1,018 cm^−1^, in the initial demineralization (Fig. 4c–d). The minor shift continued to 1,013 cm^−1^ with remineralization (demin–remin) and lastly to 1,010 cm^−1^during the acidic challenge of the remineralized surface. An abiotic acid challenge of the remineralized enamel was similar to *Rd* (gallein^pos/neg^) demineralization (Fig. 4d).

**Fig. 5.**
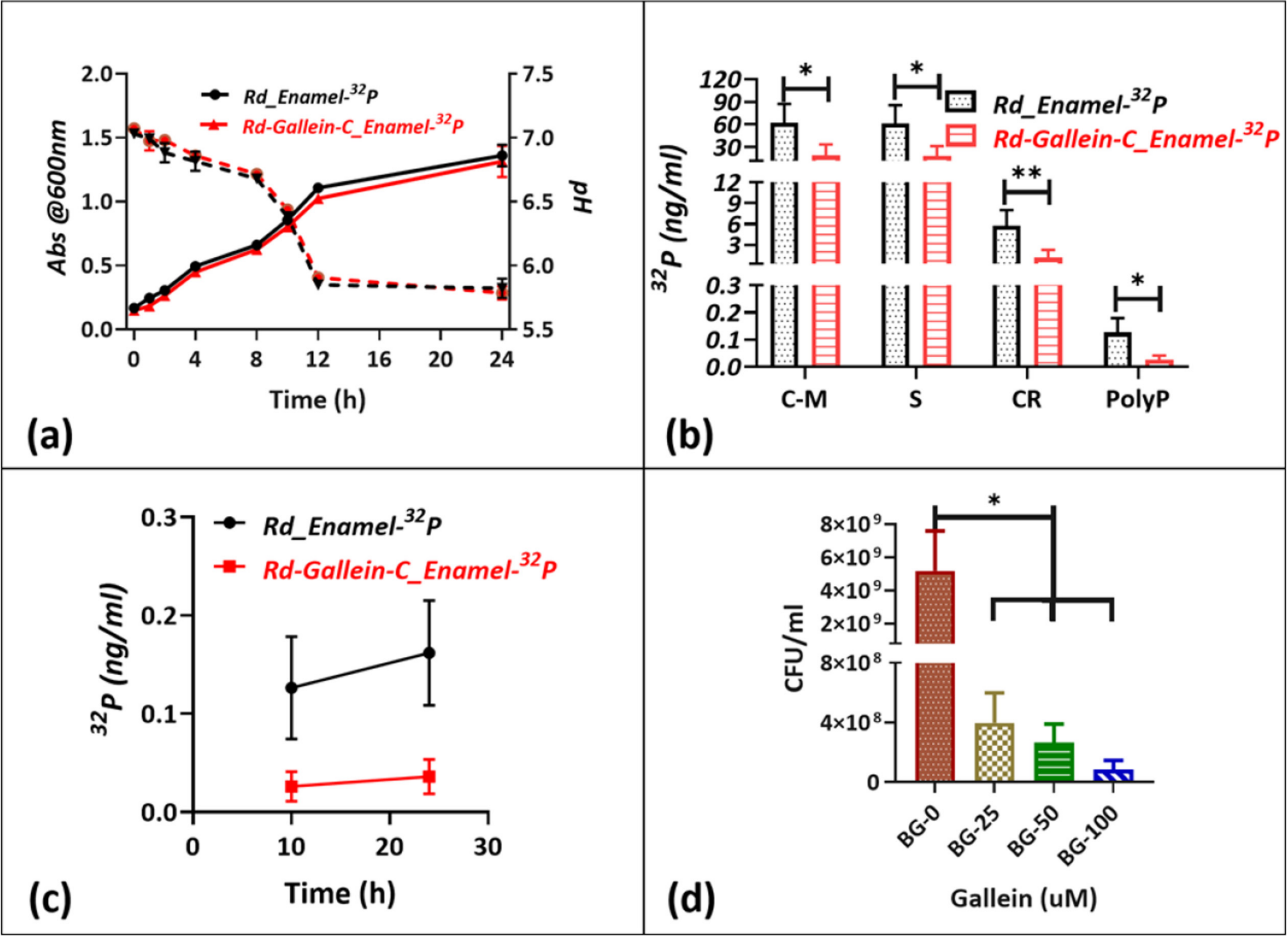
(a) Growth (solid lines) and pH (dotted) of *Rd* in BHI with gallein (red) and without (black). (b) ^32^P quantified by liquid scintillation at 10 h found within the cell-media (C-M), supernatant (S), suspended *Rd* cell pellet, with polyP extracts in samples with (red) or without (black) continuous gallein exposure. (c) The level of ^32^P found in polyP after 10 and 22 h with (red) or without (black) continuous gallein exposure. (d) Planktonic *Rd* incubated in the presence of BHI and gallein at 0, 25, 50 and 100 mM after 24 h of incubation at 37 ℃. * for *P*≤0.05, ** for *P*≤0.01.

De-Re-De samples (gallein^pos^) had a minor and statistically significant effect on planktonic growth characteristics (*P*=0.001), as measured by OD values, and acidification (*P*=0.004) of the surrounding media, during 24 h of *Rd* growth (Fig. 5a). At 24 h, the OD values of gallein^pos^ *Rd* samples were 7.45% less than gallein^neg^ samples. After logarithmic transformation of pH values to [H^+^], the gallein^pos^ *Rd* samples were calculated to have a lower [H^+^] by 7.19% compared to the [H^+^] in the gallein^neg^ samples. Examining the levels of ^32^P (ng ml^−1^) released from the De-Re-De samples at 10 h showed that gallein^pos^ had statistically significant less ^32^P found in the cell media, supernatant, *Rd* cell pellet and *Rd* polyP inclusion bodies (Fig. 5b). At 22 h, gallein^pos^ *Rd* had substantially less (22%) polyP than gallein^neg^ (Fig. 5c). Planktonic cultures of *Rd* gallein^pos^ (25, 50 and 100 mM) within the supernatant after 24 h showed a decreased ability of planktonic bacteria to be cultured directly on BHI agar plates compared with gallein^neg^ samples (Fig. 5d). Gallein inhibited the cultivability of planktonic *Rd* with a 13- to 60-fold reduction of c.f.u. ml^−1^ (25 µM to 13.03×, 50 µM to 19.58× and 100 µM to 60.57×) compared to the negative control samples.

## Discussion

This study primarily focused on controlling the uptake of inorganic phosphate by bacterial cells in an *in vitro* model and presents a novel De-Re^32^P demineralization model where ^32^P-labelled monopotassium phosphate mixed with a calcium phosphate remineralization solution repairs an initial demineralized enamel lesion. In this work, CP-OCT verified the initial formation of the demineralized lesion via higher subsurface scattering and depolarization. Sound dental enamel is nearly transparent at the SWIR wavelength of 1,310 nm, whereas demineralization results in a substantial increase in reflectivity due to crystal dissolution and a change in the refractive index when replaced by water [19]. After remineralization, the reflectivity of the enamel was near the magnitude of the original lesion, although there was a non-significant decrease in major defects that highly scatter in the enamel structure. Beyond just measuring reflectivity and depolarization, CP-OCT confirmed that there was not a high scattering precipitate that built up on the surface as seen in previous remineralization protocols [20]. While a clinical goal in remineralizing enamel lesions is to restore the enamel translucency and forward scattering component of enamel rods and eliminate the backscattering caused by porous defects [21], partially restoring some of the optical properties of the lesion requires an extended time period of 20 days in length [22]. In the current study, the half-life of ^32^P radionuclide is 14.3 days, which limits the remineralization time that would markedly decrease optical reflectivity and requires a more time-efficient remineralization protocol. The half-life of ^32^P limits the ability to use a pH cycling method to create an ideal artificial caries lesion. The acidic buffer, with high calcium and phosphate saturation, creates subsurface demineralization but causes partial surface erosion.

The main goal in the development of the De-Re^32^P-demineralized lesion model was the incorporation of ^32^P-labelled inorganic phosphate into the enamel HA to allow a consistent release profile during acidic challenge. In the present model, it was demonstrated that ^32^P-labelled phosphate release persisted over 96 h when the remineralized enamel was exposed to an acidic buffer (pH=4.5). Neutral pH water did not substantially release ^32^P-labelled P_i_. The ^32^P-labelled P was incorporated into a water-insoluble form of calcium phosphate, where acid in a buffer or acid generated from *Rd* solubilized and released the isotope from the HA crystals. This release is analogous to dental caries being caused by the excessive exposure of localized microbial acids produced by carbohydrate fermentation and/or undersaturated conditions created by phosphate uptake [23]. CP-OCT imaged the increase in scattering after the De-Remin(^32^P) enamel was exposed to *Rd* (De-Re-demin). Throughout the sequence of demineralization and remineralization, FTIR confirmed that the spectrum was nearly identical, with the exception that there was an increase in the magnitude of the signal. Importantly, after remineralization, no other types of calcium phosphate phases were identified through FTIR analysis. A region that identifies different calcium phosphate phases is near the scissors’ vibration peak of v4 PO_4_^3-^ between 525 and 610 cm^−1^ [24]. The enamel De-Remin spectra have the same dual peaks seen in the NT and demin samples. The dual-scissor vibration peaks are distinctive of HA, and the lack of additional peaks in this region rules out other calcium phosphates such as brushite, octacalcium phosphate, whitlockite and tricalcium phosphate following exposure to the remineralization process [24]. HA is the most thermodynamically stable of these calcium phosphates [25]. The experiments demonstrated that crystallinity decreased, with likely smaller deposits of individual HA crystals, through the process of demineralization–remineralization as evident from the shift in the centre peak of v1 PO_4_^3-^ at 1,031 cm^−1^ [26].

Based on the FTIR data, *Rd* was exposed to ^32^P in the form of a remineralized HA within the bovine dental enamel. To our knowledge, this is the first application of using ^32^P in a remineralization model where P_i_ from the tooth can be directly traced after bacterial exposure. As previously mentioned, Heikki Luoma established a ^32^P model to examine internalization of tooth-derived P_i_ into *S. mutans* [14–16]. This previous work involved an animal model where rats were fed the isotope during tooth formation. While this model inspired the present study, the abiotic model of remineralization with monopotassium phosphate (^32^P labelled) created a more targeted region of sustained ^32^P release when exposed to acid/bacteria while also being time- and cost-effective. In the present study, the demin–remin ^32^P was exposed to *Rd* incubated in BHI media containing 2 g l^−1^ of glucose. The resultant pH decrease from glucose fermentation solubilized the HA-bound ^32^P and was measured in the supernatant.

The experimental results confirmed the uptake of ^32^P-labelled phosphate by *Rd*. The bacteria directly imported the radionucleotide P_i_ derived from the dental enamel. Through an established and validated method of isolation and quantification [1], some of the ^32^P-labelled P_i_ was internalized by *Rd* and stored as polyP. Prior qualitative analysis has visualized intercellular polyP within oral bacteria using DAPI (4′,6-diamidino-2-phenylindole) based fluorescence microscopy [6, 7]. In the present work, ^32^P and liquid scintillation were used to quantitatively measure P_i_ derived directly from enamel within polyP and the effects of gallein. *Rd* used the P_i_ derived directly from the tooth for the benefit of the bacterium’s fitness. PolyP has a virulence role via modulating stress response, survival and viability [27]. Exposure of *Rd* to 25 mM gallein caused less than 10% reduction of planktonic growth and pH compared to untreated controls. The effect of gallein against polyP production was more substantial, and this affected the bacterium’s viability when plated onto agar plates. This is likely due to gallein’s effect on polyP kinases. Inhibition of polyP kinases through genetic alteration or inhibition with gallein has demonstrated deficiencies in stress response [28, 29]. While this approach is novel in the context of oral cariology, several different investigations have shown that polyP can directly affect the virulence of other bacteria such as *Mycobacterium tuberculosis*, *Shigella flexneri*, *Pseudomonas aeruginosa* and *Salmonella enterica* [28, 30–32]. There are several additional oral clades besides *Rothia* that have ppk/ppk genes, such as *Lactobacillaceae*, *Corynebacterium* and *Neisseria* [6, 7, 33]. A limitation of the present study is that the investigation was limited to *Rd*. The oral microbiome contains additional species with variable genetic potential to synthesize and regulate polyP. More work is needed to investigate the breadth of polyP producers that may take up high amounts of extracellular P_i_.

Another limitation of the present study is its use of a planktonic model that accounts only for salivary bacteria [34, 35]. A ^32^P uptake model with planktonic bacteria was used to standardize suspension volumes across conditions and proved to be reproducible. Biofilm sampling in a ^32^P model introduces potential variability to the assay, including variability in sample volume. Additionally, biofilm cultivation with radioisotope labelling increases radiation safety risk and disposal needs. Future studies need to address these biofilm-related issues with advanced mechanized methods. It would be advantageous to examine a biofilm model, such as plaque-derived oral microcosms [36]. While both salivary (planktonic) and plaque (biofilm) bacteria generate acid from fermentable carbohydrates and contribute to caries risk, plaque bacteria are uniquely capable of producing extensive demineralization [37]. It has previously been shown that bacteria in biofilms also take up phosphate and utilize polyP, like planktonic bacteria [6]. But little is known about the magnitude of this uptake, especially in multispecies biofilm models, and whether P uptake both improves biofilm stress response and reduces localized phosphate concentrations at the enamel interface, which could affect mineral dissolution.

More work is also necessary to understand localized effects of gallein on dental soft tissue since gallein has been shown to have anti-tumour effects and reduce platelet activity [38, 39]. Gallein is noncytotoxic up to 100 µM concentration in mammalian cells (human embryonic kidney 239 T cells) with limited off-target effects on other human kinases [40]. Additionally, gallein can reduce acute inflammation [41]. Despite the potential for low toxicity, more work is needed to understand the breadth of chemotactic signalling pathway inhibition and chronic exposure effects. The safety and potential application as an oral hygiene additive needs further investigation. Future studies may consider the use of gallein as an additive in bioactive dental materials placed inside a restored tooth. These studies should examine gallein’s effect on polyP producers within mature multispecies biofilms. Gallein has the potential to suppress the viability of bacteria that penetrate along the material interface and use P_i_ derived from demineralization during secondary caries as an energy storage and stress response molecule.

## Supplementary material

10.1099/jmm.0.002192 Supplementary Material 1.

## Abbreviations

ATR-FTIR: attenuated total reflectance Fourier transform infrared
BHI: brain heart infusion
CP-OCT: cross-polarization optical coherence tomography
HA: hydroxyapatite
NT: no treatment
^32^P: phosphorus-32
P: phosphorus
P_i_: orthophosphate
polyP: polyphosphate
*Rd*: *Rothia dentocariosa*
SWIR: shortwave infrared
