# Supplementary material for: Ex vivo inhibition of enamel phosphate uptake in Rothia dentocariosa
Source: J Med Microbiol. 2026 Aug 7;75(8):002192. doi: 10.1099/jmm.0.002192 (PMC13450940; doi:10.1099/jmm.0.002192)
Supplement: Supplementary file 1 [file jmm-75-02192-s001.pdf]

Supplemental Material

Enamel Phosphate Uptake By *Rothia dentocariosa* and Gallein Inhibition

Dhiraj Kumar[1*], Robert S. Jones[2*], Beverly E. Flood[3], and Jake V. Bailey[3]

1 Department of Diagnostic & Biological Sciences, University of Minnesota, Minneapolis, MN 55455.
2 Department of Developmental & Surgical Sciences, University of Minnesota, Minneapolis, MN 55455.
3 Department of Earth and Environmental Sciences, University of Minnesota, Minneapolis, MN 55455, USA.

*Co-correspondence: Dhiraj Kumar: kumard@umn.edu; Robert S. Jones rsjones@umn.edu

Materials and Methods

Reflectivity and Optical Coherence Tomography

Bovine teeth were embedded in a polymer resin (ethylene-vinyl acetate (EVA), polyesters, polyethylene, polyamide, polyolefin and ethylene-methyl acrylate (EMA)) block using the 1cm x 1cm x 1cm, silicon mold. The exposed enamel section of size 4 x 4 mm was embedded carefully in the middle of the resin block exposing only the top enamel part. The surface and subsurface enamel reflectivity in the shortwave infrared (SWIR) wavelength region was used to non-destructively assess the extent of the *in vitro* demineralization and remineralization prior to, and after, exposure to *Rd* or a demineralizing solution. To measure enamel reflectivity, a SWIR cross polarization optical coherence tomography (CP-OCT) system (IVS-2000, Santec, Japan), with a 30 kHz swept rate continuous wavelength scanning laser, was used to obtain tomographic images of the bovine enamel samples. Cross-polarization, which uses incident parallel polarized light and measures backscattered light in the orthogonal axis, was utilized to minimize the effects of surface reflection on bulk enamel crystal reflectivity (Fried et al. 2002). The center wavelength of the SWIR system was 1310 nm with a bandwidth of 104 nm and an axial resolution of <10µm in enamel. The lateral resolution of the SWIR CP-OCT system was 80 µm that is based on the focusing optics (Lenton et al. 2012). Full description and schematics of the system and discussion of CP-OCT

imaging are described elsewhere (Liu, B and Brezinski, M 2007; Lenton et al. 2012). Acquired reflectivity values were converted into logarithmic false color scale (dB) tomograms using a custom Matlab script (Mathworks, Natick, MA). Demineralization was assessed at two thresholds corresponding to moderate and major optical reflectivity changes. Moderate reflectivity changes were assessed at 2 dB above background intensity but below 20 dB. Major reflectivity changes were at and above 20 dB. A reflectivity change at 2 dB above baseline is nearly a 50% increase in scattering and 20 dB change is close to a 100-fold increase in reflectivity.

Radionuclide Phosphate Analysis and PolyP isolation

On day 4, cell-media suspensions exposed to the radioisotope labeled remineralized enamel were analyzed for ^{32}P activity at 10 and 22 hr time points. The cell-media broth (10 ml) was divided into four sets of analysis that examined the cell-media, supernatant, cell resuspension, and polyP isolation. From each set, 1.0 ml suspension was mixed with 10.0 ml scintillation liquid and homogenized for collecting scintillation counts (SC), with a collection time of 60 seconds. For cell-media SC counts (n=6), the sample solution was homogenized, and 1 ml solution was mixed with 10 ml cocktail solution for SC reading. For cell pellet count and supernatant count, the 5.0 ml cell-media suspension was centrifuged at 5000 rpm in a 10 ml centrifuge tube for 5 min at 4°C. The supernatant was carefully collected in a clean 10ml centrifuge tube and the cell pellet was redispersed-homogenized in 5.0 ml fresh BHI media. From each sample tube (either supernatant or cell pellet redispersed in BHI media) 1 ml sample was mixed with 10.0 SC cocktail for SC measurement. From the cell pellet in fresh BHI media, 1.0 ml cell mixture was used for the isolation of polyP on day 6 as described previously and based on a validated protocol for isolating polyP (Bru et al. 2016; Kumar et al. 2023). PolyP isolation method began with the dispersion of *Rd* cells by vortexing them in 400µL of sodium acetate/EDTA buffer and 40µL 10% SDS solution. 300µL of phenol was then added and the mixture was incubated for 5 min at 65°C followed by snap cooling in ice. After the addition of 300µL of chloroform, the mixture was vortexed followed by centrifugation (13000g/5 min/room temperature). The phenol-chloroform extraction created a protein

precipitate, and the overlaying polar layer devoid of precipitates was removed. Then homogenization was repeated with fresh chloroform (350 μL) followed by 5 min centrifugation. The polar aqueous phase was pipetted into a new 2 ml tube with 10 μL of DNAse and 10 μL RNAse solutions. The RNAse and DNAse solutions were made from a 50 mM sodium acetate with 10 mM EDTA buffer solubilizing 10 mg/ml of RNAse and DNAse (Millipore Sigma, Burlington, MA, USA). The sample mixture was kept at 37°C using a temperature-controlled incubator for 2h. 1 ml of 100% ethanol, which was pre-cooled at -20°C, was added to the tube along with 40 μL 3M sodium acetate and 10 mM of EDTA. Overnight incubation at -20°C followed with next day centrifugation (13000 g/15 minute/4°C). Supernatant was removed from the precipitated PolyP, which was re-solubilized in 400 μL 70% ethanol. The solutions were vortexed, centrifuge (13000g/5 minutes/4°C), and the supernatant was decanted carefully. The isolated polyP was redispersed in 1.0ml H_2SO_4 (4.2M) and vortexed vigorously for 10-15 minutes to homogenize, followed by carefully transferring the volume in 10.0 ml scintillating cocktail solution for scintillation count reading immediately.

Supplemental Material Figure 1. Using ^{32}P radionuclide, in the form of monopotassium phosphate (1 millicuries, mCi), beta-emission was measured by a liquid scintillation counter (LS6500, Beckman Coulter, Brea, CA, USA). The degree of beta emission in ^{32}P decays over time. Since beta emission is specific, predictable, and reproducible over time, calibration curves were created and correlated ng/ml with scintillation counts that measure the beta emission. Standardize calibration curves were created by measuring KH_2PO_4 (ng/ml) against scintillation counts over at day 0, 4,5,6. This provided the ^{32}P beta-emission decay per time.

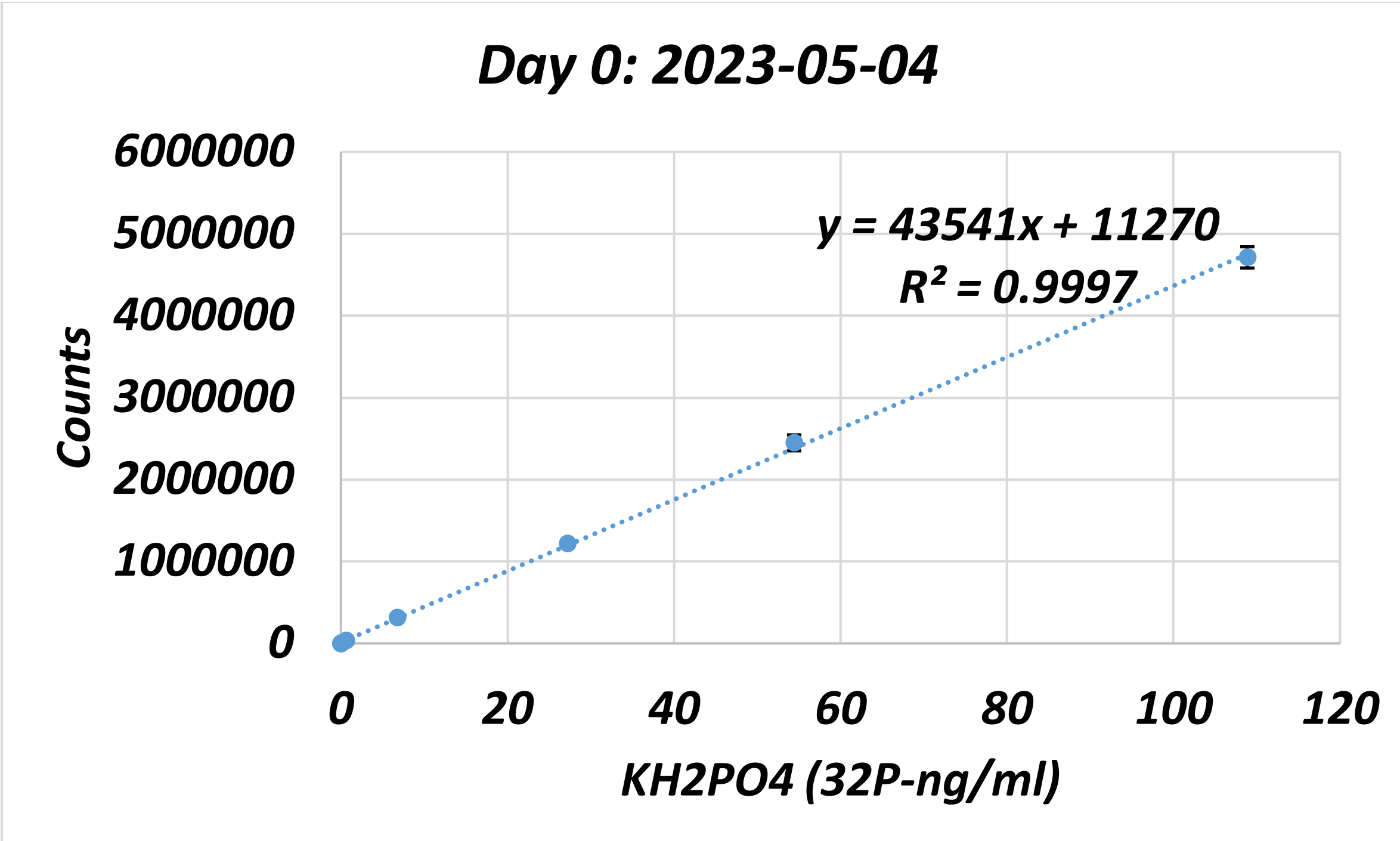

Day 0: 2023-05-04
y = 43541x + 11270
R² = 0.9997
Counts
6000000
5000000
4000000
3000000
2000000
1000000
0
0
20
40
60
80
100
120
KH2PO4 (32P-ng/ml)

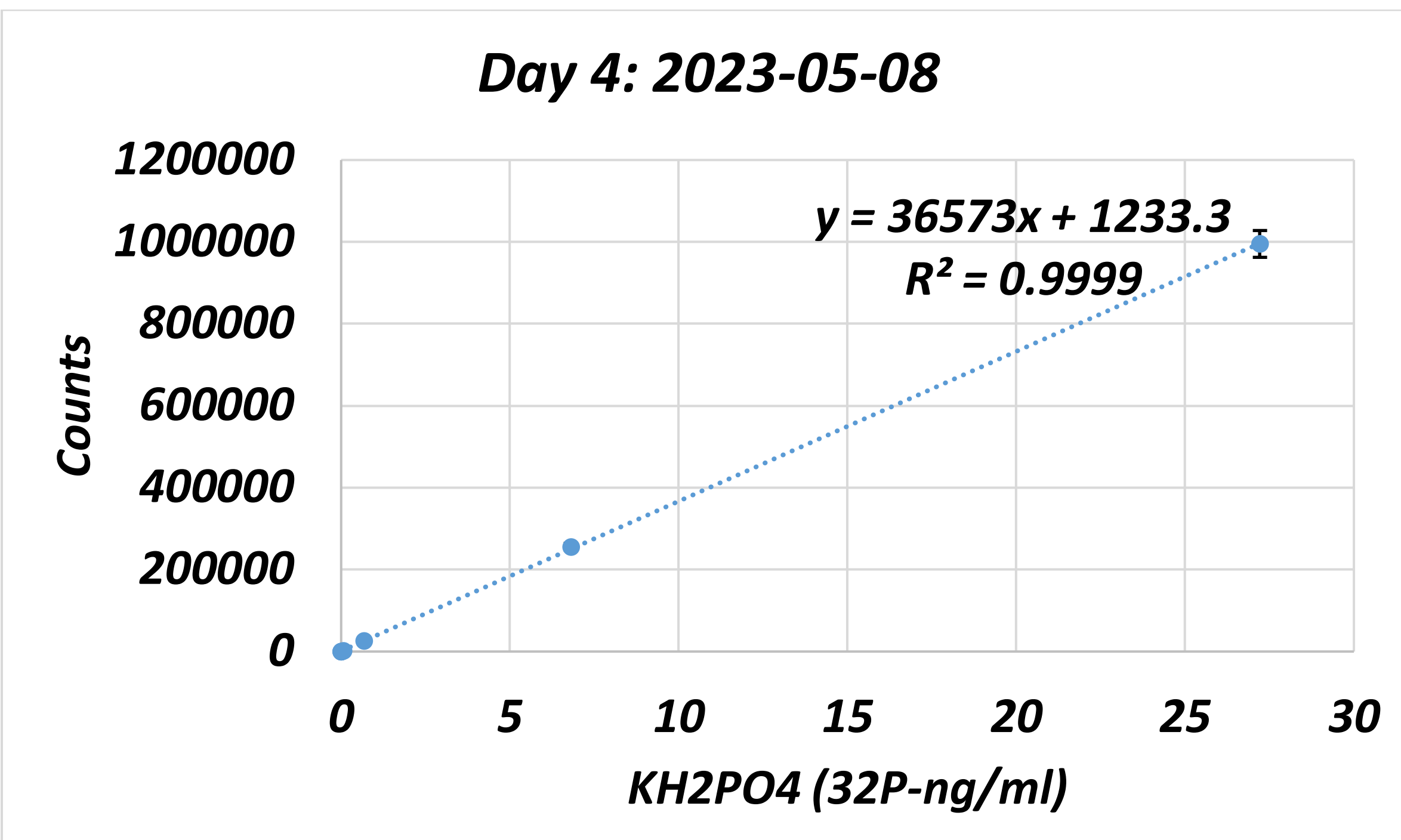

Day 4: 2023-05-08
y = 36573x + 1233.3
R² = 0.9999
Counts
1200000
1000000
800000
600000
400000
200000
0
0
5
10
15
20
25
30
KH2PO4 (32P-ng/ml)

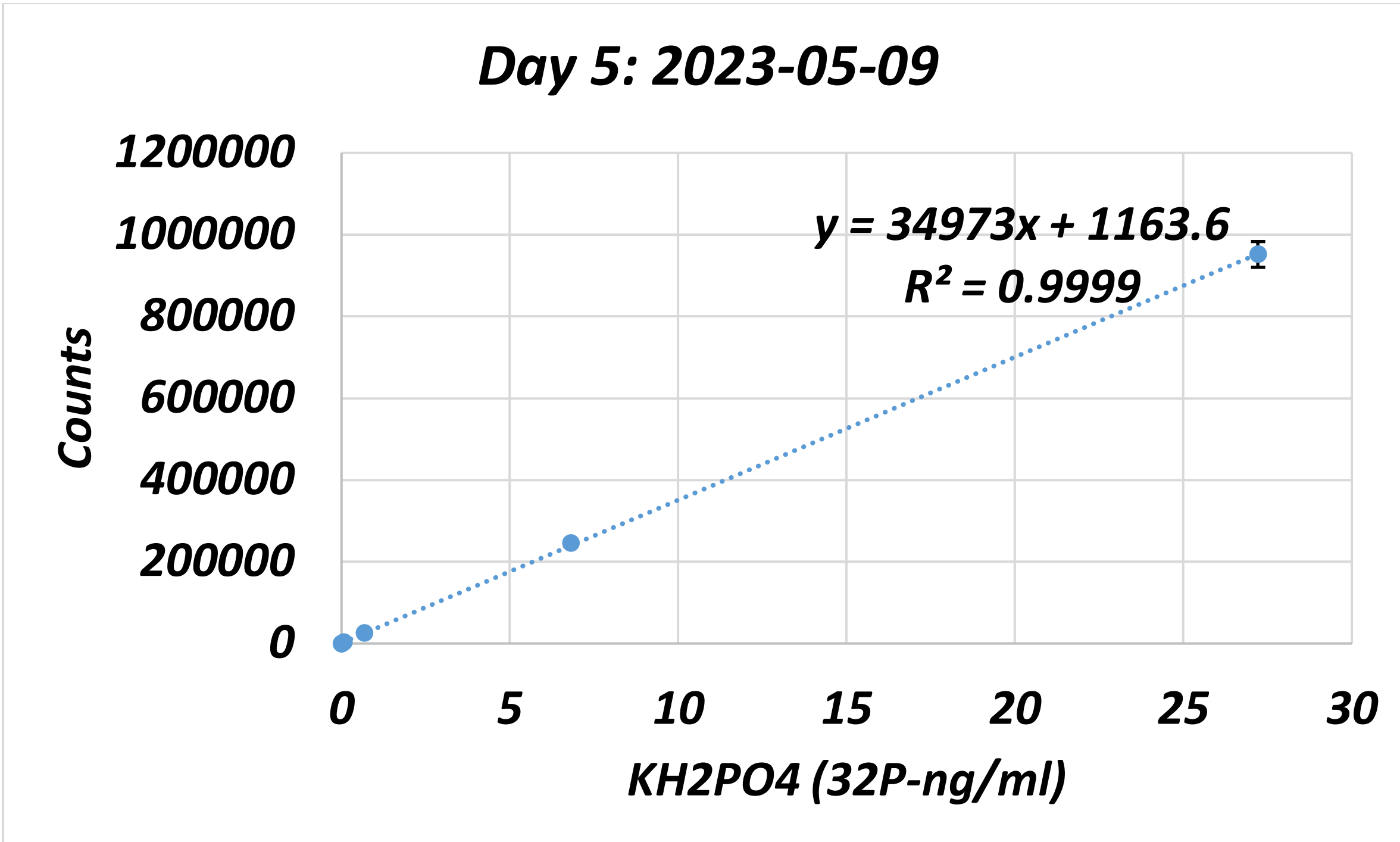
Day 5: 2023-05-09
y = 34973x + 1163.6
R² = 0.9999
Counts
1200000
1000000
800000
600000
400000
200000
0
0
5
10
15
20
25
30
KH2PO4 (32P-ng/ml)

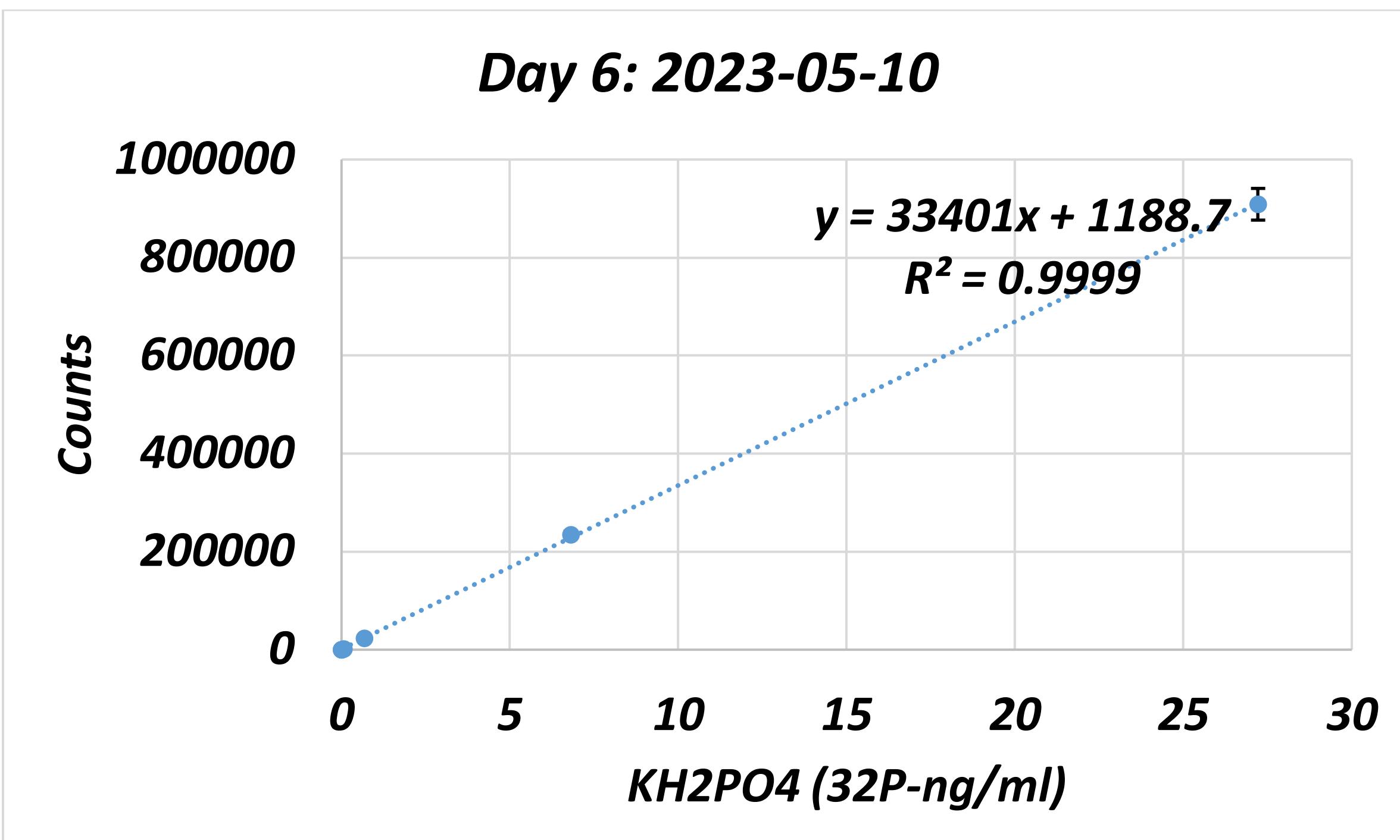
Day 6: 2023-05-10
y = 33401x + 1188.7
R² = 0.9999
Counts
1000000
800000
600000
400000
200000
0
0
5
10
15
20
25
30
KH2PO4 (32P-ng/ml)

Supplemental Material Figure 2 The full FTIR spectra of the no treatment control enamel (NT) were similar to subsequent demineralization and remineralization challenges with the exception that the signal intensity increases Peaks at 3200-3450 cm^{-1} indicate hydroxyl band stretching. Bands from 1400 to 1550 cm^{-1} are characteristic of v3 $C0_3^{2-}$ stretching and elongation. The large band at 1030 cm^{-1} corresponds to the stretching vibration of v1 P-O with scissor vibrations of v4 PO_4^{3-} at 525-610 cm^{-1} (Figure 4B).

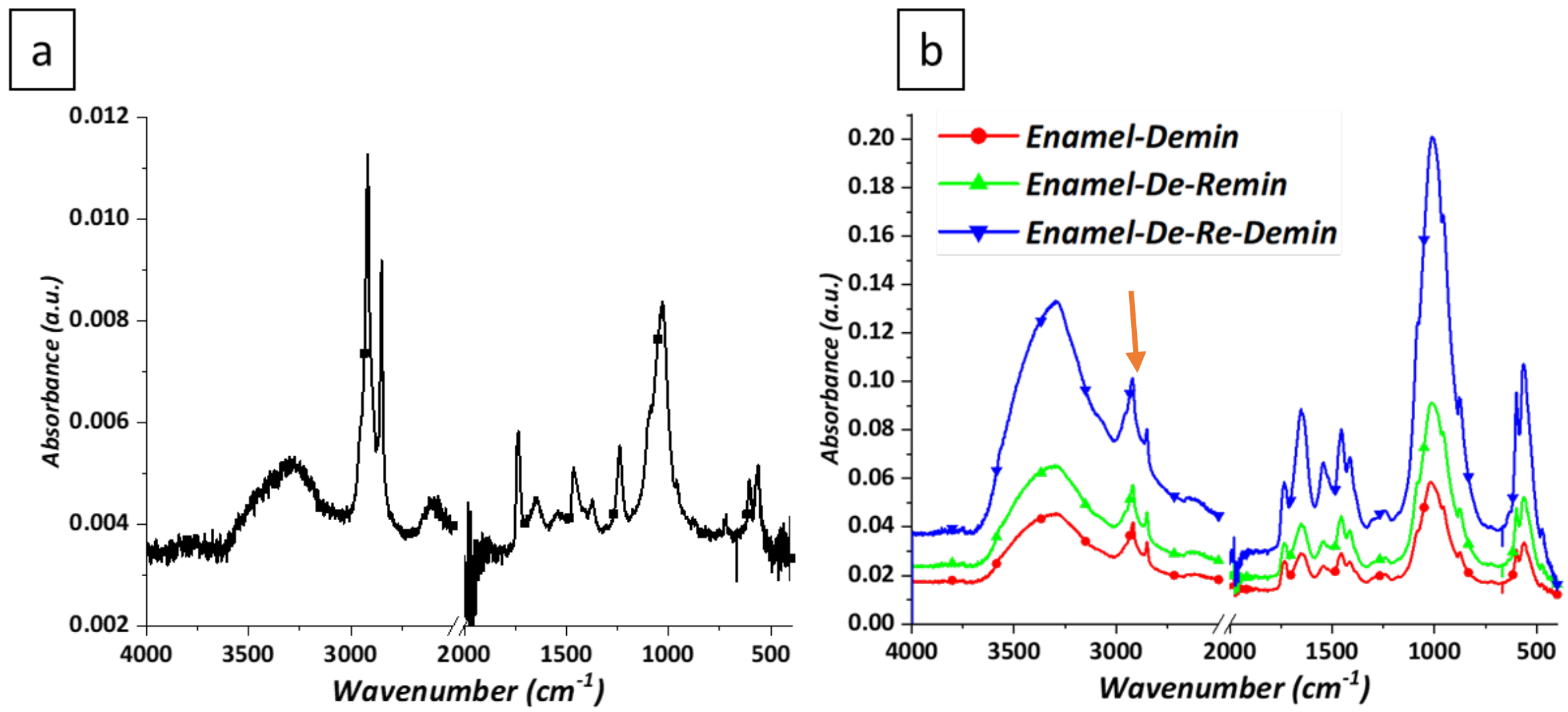

Additional References

Bru S, Jiménez J, Canadell D, Ariño J, Clotet J. 2016. Improvement of biochemical methods of polyP quantification. Microb Cell. 4(1):6–15.

Fried D, Xie J, Shafi S, Featherstone JDB, Breunig TM, Le C. 2002. Imaging caries lesions and lesion progression with polarization sensitive optical coherence tomography. J Biomed Opt. 7(4):618–627.

Kumar D, Mandal S, Bailey JV, Flood BE, Jones RS. 2023. Fluoride and gallein inhibit polyphosphate accumulation by oral pathogen Rothia dentocariosa. Letters in Applied Microbiology. 76(2):ovad017.

Lenton P, Rudney J, Chen R, Fok A, Aparicio C, Jones RS. 2012. Imaging in vivo secondary caries and ex vivo dental biofilms using cross-polarization optical coherence tomography. Dental materials. 28(7):792–800.

Liu, B, Brezinski, M. 2007. Theoretical and practical considerations on detection performance of time domain, Fourier domain, and swept source optical coherence tomography. Journal of Biomedical Optics. 12(4):044007.
